# The potential neuroprotective effects of cannabinoids against paclitaxel-induced peripheral neuropathy: *in vitro* study on neurite outgrowth

**DOI:** 10.3389/fphar.2024.1395951

**Published:** 2024-06-12

**Authors:** Ioana Creanga-Murariu, Leontina-Elena Filipiuc, Maria-Raluca Gogu, Mitica Ciorpac, Carmen Marinela Cumpat, Bogdan-Ionel Tamba, Teodora Alexa-Stratulat

**Affiliations:** ^1^ Advanced Research and Development Center for Experimental Medicine (CEMEX), Iasi, Romania; ^2^ Grigore T. Popa University of Medicine and Pharmacy, Iasi, Romania; ^3^ Oncology Department, Regional Institute of Oncology, Iasi, Romania; ^4^ Clinical Rehabilitation Hospital, Cardiovascular and Respiratory Rehabilitation Clinic, Iasi, Romania

**Keywords:** neuropathic pain, cannabis, THC, CBD, antalgic, palliative care, chemotherapy, cellular model

## Abstract

**Introduction:** Chemotherapy-induced peripheral neuropathy (CIPN) is a shared burden for 68.1% of oncological patients undergoing chemotherapy with Paclitaxel (PTX). The symptoms are intense and troublesome, patients reporting paresthesia, loss of sensation, and dysesthetic pain. While current medications focus on decreasing the symptom intensity, often ineffective, no medication is yet recommended by the guidelines for the prevention of CIPN. Cannabinoids are an attractive option, as their neuroprotective features have already been demonstrated in neuropathies with other etiologies, by offering the peripheral neurons protection against toxic effects, which promotes analgesia.

**Methods:** We aim to screen several new cannabinoids for their potential use as neuroprotective agents for CIPN by investigating the cellular toxicity profile and by assessing the potential neuroprotective features against PTX using a primary dorsal root ganglion neuronal culture.

**Results:** Our study showed that synthetic cannabinoids JWH-007, AM-694 and MAB-CHMINACA and phytocannabinoids Cannabixir^®^ Medium dried flowers (NC1) and Cannabixir^®^ THC full extract (NC2) preserve the viability of fibroblasts and primary cultured neurons, in most of the tested dosages and time-points. The combination between the cannabinoids and PTX conducted to a cell viability of 70%–89% compared to 40% when PTX was administered alone for 48 h. When assessing the efficacy for neuroprotection, the combination between cannabinoids and PTX led to better preservation of neurite length at all tested time-points compared to controls, highly drug and exposure-time dependent. By comparison, the combination of the cannabinoids and PTX administered for 24 h conducted to axonal shortening between 23% and 44%, as opposed to PTX only, which shortened the axons by 63% compared to their baseline values.

**Discussion and Conclusion:** Cannabinoids could be potential new candidates for the treatment of paclitaxel-induced peripheral neuropathy; however, our findings need to be followed by additional tests to understand the exact mechanism of action, which would support the translation of the cannabinoids in the oncological clinical practice.

## 1 Introduction

Chemotherapy-induced peripheral neuropathy (CIPN) is a common side effect of anticancer drugs, with the incidence varying from as low as 12.1% to as high as 96.2% (Masocha, 2018). Some chemotherapy drugs are known to be associated with a high incidence of CIPN, such as platinum derivates, vinca alkaloids, Bortezonib, or Thalidomide. Taxanes, in particular, are associated with a high incidence of CIPN, with paclitaxel (PTX) responsible for more than 70% of the cases (Seretny et al., 2014). Moreover, a significant proportion of patients developing CIPN after PTX have persisting symptoms for up to 1 year after the end of the treatment (Seretny et al., 2014; Burgess et al., 2021), most often reported as paresthesia, loss of sensation, and dysesthetic pain in the feet and hands with a “stocking and glove” distribution (Staff et al., 2020). Particularly noteworthy for the clinical practice is that due to the disturbing and persistent symptoms patients perceive, the physician is often forced to reduce the dosage of Paclitaxel, even though reductions below 85% are known to decrease survival rates significantly (Manfredi and Horwitz, 1984).

The pathology of CIPN is complex and still poorly understood. However, the direct effects of the chemotherapy on the peripheral nerves are responsible for the patient’s painful sensation. PTX’s primary mode of action is hyper-stabilizing the microtubules, preventing the normal cycles of microtubule depolymerisation and repolymerization within the cytoskeleton (Manfredi and Horwitz, 1984). PXT’s effects vary between different types of cells, such as tumoral cells, where the tubulin polymerisation hinders the formation of the mitotic spindle, causing the arrest of the cells in the G2/M-phase of the cell cycle, whereas in non-dividing cells, such as neurons, paclitaxel-induced tubulin polymerisation is thought to interfere with axonal transport, causing peripheral neuropathy (Manfredi and Horwitz, 1984). Besides the effects on the microtubules, the neurotoxicity of PTX could also be exerted by other mechanisms, such as mitochondrial dysfunction, immune response induction, and calcium ion disruption (Zajączkowska et al., 2019; Desforges et al., 2022).

The current treatment options for CIPN evolve around painful symptom control. The oncology practice guidelines developed by the European Society of Medical Oncology (ESMO) and the American Society of Clinical Oncology (ASCO) advise the prescription of anticonvulsants, tricyclic antidepressants, serotonin-norepinephrine reuptake inhibitors and opioids (Jordan et al., 2020; Loprinzi et al., 2020). The clinical management of CIPN is difficult, given the fact that the efficacy of the named medications is limited, varies greatly between the patients and can cause serious side effects (Jordan et al., 2019). Nevertheless, the medication aims only to decrease the symptom intensity and doesn’t provide a curative approach to the pain. Current practices for the prevention of high-grade CIPN is to provide special attention to the onset of painful symptoms when administering the neurotoxic treatment in order to reduce the chemotherapy dose if necessary or to change the class of medication (Hausheer et al., 2006). However, this approach is not ideal, given the fact that reductions below 85% of the effective antitumoral dosages are known to decrease survival rates significantly (Manfredi and Horwitz, 1984). On the other hand, targeting neuroprotection as an alternative option for preventing and treating CIPN is attractive, however it is a strategy that is not yet included in the guidelines (Loprinzi et al.).

The need for new drugs in this setting shifted the attention of clinicians and researchers to cannabinoids, as they have been studied for their neuroprotective activity in a range of diseases and pathological conditions, including Alzheimer’s disease, Parkinson’s, multiple sclerosis, dementia and nevertheless neuropathic pain of various etiologies (Pacher and Kunos, 2013). Endogenous or exogenous cannabinoids interact with the endocannabinoid system, heterogeneously present in different structures of the central and peripheric nervous system, including essential regions of pain processing, such as the spinal cord, thalamus, amygdala and dorsal root ganglions (DRGs) (Finn et al., 2021). A necessary part of the endocannabinoid system is represented by the cannabinoid receptors 1 and 2 (CB1R and CB2R), present in pain pathways from the peripheral sensory nerve endings up to the brain (Barinaga, 2001). CB1R are mainly expressed throughout the central nervous system (CNS), in key areas responsible for pain transmission. They are involved in the attenuation of synaptic transmission and modulation of another neuronal mechanism due to their expression on the primary afferent neurons (Ibrahim et al., 2003). The activation of CB1R from primary afferent neurons, DRGs and brain regions involved in pain processing is associated with a decrease in neuronal excitability and a dampening of neurotransmission (Brown and Winterstein, 2019). Also, the activation causes a decrease in the release of neurotransmitters such as dopamine and GABA, conducting to a neuroinhibitory state (Piomelli et al., 2000). Contrary, CB2R expression is non-neuronal and is found in glia, immune cells and peripheral tissues. For this reason, CB2 selective ligands could modulate pain by inhibiting the release of proinflammatory factors by non-neuronal cells located near nociceptive neuron terminals (Guindon and Hohmann, 2009; Vera et al., 2013).

Exogenous ligands of the cannabinoid receptors are either naturally sourced, derived from plants belonging to the *Cannabis* genus or synthetically engineered to mimic the structure and function of some phytocannabinoids (PCs) (Creanga-Murariu et al., 2023). The first discovered were the PCs, of which the most known active molecules are Δ9-tetrahydrocannabinol (Δ9-THC) and cannabidiol (CBD) (Mechoulam and ShvoHashish, 1963). Each cannabis plant has a vast assortment of active compounds that vary in composition, concentration and ratio depending on environmental factors, genetic background and even morpho-spatial position of the plant (Danziger and Bernstein, 2021). There are more than 100 lipid-soluble molecules within each cannabis plant, which could explain the versatility of medicinal use (analgesic, anticonvulsant, antispasmodic, diuretic, expectorant etc. (Zuardi, 2006)) and the unique feature of synergistic effects (the “entourage effect”) with the cannabinoids (Filipiuc et al., 2021). Synthetic cannabinoids (SCs) are compounds functionally similar to PCs but entirely different in composition, lacking the vast combinations of active molecules. They were initially designed as ligands for the identification of endogenous cannabinoid system, particularly the CRs. These chemically engineered drugs mimic the PC’s mode of action by acting on the same receptors. However, they are more potent due to their activity as full agonists and their higher affinity for cannabinoid receptors (Tamba et al., 2020). Due to the intense effects, SCs entered the black market as recreational drugs, administered incorrectly, with important side effects. As such, acute, severe or unpredictable side effects have been reported following SC abuse, and hospital admission rates are consistently higher for SC use than for natural cannabinoid consumption (Tamba et al.). SCs come with the advantage of predictable pharmacokinetics and pharmacodynamics as their synthetization is usually conducted in established laboratories. On the other hand, cannabis plants can come from farmers that don’t always subject to the regulation of Good Manufacturing Practices to ensure the standardisation of the product, which can conduct the growing of plants genotypically and phenotypically different than the originals (de Souza et al., 2022).

The prevalence of cancer patients using any form of cannabinoids was between 25% and 40%, as reported by a recent study (Pergam et al., 2017). The drugs were either physician-prescribed or illicitly procured, and the majority of the patients reported using them to manage pain symptoms (Tringale et al., 2019). Although widespread, one needs to be aware of the multitude of side effects, especially the increased risk of psychosis, cognition impairment, sedation, and cardiovascular and pulmonary function alteration (Creanga-Murariu et al., 2023). These side effects are highly dependent on the ratio between active molecules, potency, dosages, age of the patients, naïve vs. experienced users and acute vs. chronic exposure (Creanga-Murariu et al., 2023). Consequently, the scientific field must keep up with every new candidate by thoroughly testing each one’s toxicity and efficacy to distinguish between beneficial and life-threatening effects.

During the last years, cannabinoids have been studied as a valuable option to successfully modulate the endocannabinoid system in preclinical models with the outcome of neuroprotection, bringing an alternative for cancer patients suffering from neuropathic pain (Rahn et al., 2007; Yamamoto et al., 2008; Scott et al., 2014; Romero-Sandoval et al., 2017). Fewer studies have been conducted in the human population. However, the patients showed a decrease in the symptom intensity when cannabis was added to their standard therapy (Lynch et al., 2014; Creanga-Murariu et al., 2023).

As pain is the most common and troublesome symptom of CIPN and nerve dysfunction is an important pillar observed in the pathogenesis, it leads to the hypothesis that neuropathic pain is, in fact, a symptom of neurodegeneration (Bordet and Pruss, 2009). Nonetheless, suppose neurodegeneration is a mechanism responsible for the onset of neuropathic pain. In that case, it seems logical to consider neuroprotective drugs as tools to prevent the onset, control progression, or even reverse the nerve damage leading to neuropathic pain. However, the clinical practice still lacks efficient strategies for the prevention of neuropathy, hence pain, in the case of patients exposed to known neurotoxic chemotherapy. Only a few drugs have been explored with the aim of preventing or delaying the onset of CIPN (e.g., Acetyl-L-carnitine, alpha-lipoic acid, or cryotherapy). However, no firm recommendation for clinical use is endorsed by the guidelines (Loprinzi et al., 2020).

In the present study, we are motivated by the high burden of cancer patients experiencing CIPN, the poorly understood pathogenesis of neuropathic pain, the absence of efficient treatment and the attractive potential of cannabinoids as new analgesic agents for cancer patients. Therefore, we aim to screen three SCs JWH-007, AM-694, MAB-CHMINACA and two phytocannabinoids, Cannabixir^®^ Medium dried flowers (NC1) and Cannabixir^®^ THC full extract (NC2), which have not been tested before with this indication, for their general toxicity and efficacy for neuroprotection, thus analgesia. Using a primary dorsal root ganglion neural culture, our experiment design will assess neuroprotection regarding overall neuronal morphology and neurite length. We chose the model given that unbalanced synaptic communication in the dorsal horn of the spinal cord is one of the leading causes of neuropathic pain consolidation (Campos et al., 2021). We hypothesise that cannabinoids offer neuroprotection for neurons directly exposed to PTX and could be potentially translated to the clinic as new agents for the prevention or delay of the CIPN.

## 2 Materials and methods

### 2.1 Materials

#### 2.1.1 Drug formulation

Synthetic cannabinoids JWH-007, AM-694, and MAB-CHMINACA were purchased from Cayman Chemical (Cat # 155471-10-6; 335161-03-0; 22047; Ann Arbor, MI, United States). Natural cannabinoids, Cannabixir^®^ Medium dried flowers (NC1) was sourced from Cansativa GmbH (Mörfelden-Walldorf, Germany); Cannabixir^®^ THC full extract (NC2) was sourced from FYTA Company B.V., (Netherlands). The compounds were dissolved in DMSO (0.1%). An antineoplastic agent from the taxane group, semisynthetic Paclitaxel 98% purity, was purchased from Sigma Aldrich (Cat # 33069-62-4 *,* Darmstadt, Germany).

#### 2.1.2 Chemicals

For the toxicity studies, V79 cell line was purchased from ATCC (Cat # CCL-93; Virginia, United States); substrate 3-(4,5-dimethylthiazol-2-yl) 2,5 diphenyl tetrazolium bromide (MTT powder, Cat # M5655), Dimethylsulfoxide (DMSO, Cat # 67-68-5), Dulbecco’s Modified Eagle Medium (DMEM, Cat # D6429) and Fetal Bovine Serum (FBS, Cat # F7524) were purchased from Sigma-Aldrich (Saint Louis, United States). For efficacy studies (primary DRG neurons culture), dissection and culture media (RPMI 1640, Cat # R8758; Neurobasal A* Cat #10888022), supplements (Penicillin-Streptomycin Cat # P4333, B27* Cat # 17504044, Glutamax* Cat # 35050061), enzymes (Collagenase Cat #C9697, Trypsin EDTA 0.25%* Cat # 25200056, DNAse* Cat # 18047019, Trypsin Inhibitor Cat T6522), Percoll (Cat # P1644), Poly-D-Lysine (Cat # P3513) and Laminin (Cat # L2020) were also purchased from Sigma-Aldrich (Saint Louis, United States) and ThermoFisher Scientific* (Waltham, MA, United States) respectivley.

Animal anaesthesia: Isoflurane 2% (ISOFLUTEK 1000 mg/g inhalation vapour, liquid) used for animals’ anaesthesia was obtained from Laboratorios KARIZOO, S.A. (Caldes de Montbui, Spain) and used according to literature (Marquardt et al., 2018).

#### 2.1.3 Animals for dorsal root ganglion harvesting

Swiss Albino female mice, 8–12 weeks old, 20–40 g weight were kept in a controlled environment (20°C ± 4°C room temperature, 50% ± 5% relative humidity, and a 12 h artificial light-dark cycle, 07.00 a.m./07.00 p.m.), in individually ventilated cages (IVCs), with *ad libitum* access to food and water, in the CEMEX animal research facility of the Grigore T. Popa University of Medicine and Pharmacy, Iasi, Romania. The experimental study was carried out in compliance with European Directive 2010/63/EU. It was authorised by the university’s Research Ethics Committee (no. 47/17.02.2021) and approved by the Romanian National Sanitary Veterinary and Food Safety Authority (no. 34/07.04.2021).

### 2.2 Methods

#### 2.2.1 Fibroblast toxicity of the cannabinoids

Preliminary toxicity tests were performed using fibroblast culture (V79 cell line). In each well of a 96-well plate, 3000 cells were incubated for 24 h at 37°C, 5% carbon dioxide. Serial dilutions (5 µM, 10 μM, 15 μM, 20 μM, and 25 µM) of each cannabinoid was administered for another 24 h. The growth media of the controls contained a similar concentration of DMSO (0.1%), which was used for treatment formulation to exclude any solvent effects on cell viability. We chose our dosages based on similar work published (Cooper and Goldstein, 1976; Miyato et al., 2009; Kosgodage et al., 2018; Saliba et al., 2019; Griffiths et al., 2021; Kim et al., 2021). Treated cell lines were examined microscopically to detect morphological changes or detached cells.

Cell viability was evaluated using the MTT assay, which involves the conversion of the water-soluble yellow dye MTT [3-(4,5-dimethylthiazol-2-yl)-2,5-diphenyltetrazolium bromide] to insoluble purple formazan by the action of mitochondrial lactate dehydrogenase enzymes (LDH). Further, DMSO was used to dissolve the insoluble purple formazan product into a coloured solution. Treated cells were washed once with phosphate-buffered saline (pH 7.2 0.2), and residual live cells were stained with 0.5% MTT solution. After 2 hours of incubation with the dye, MTT was discarded, and the newly formed intra-cytoplasmic MTT formazan crystals were dissolved using DMSO. The experiment was conducted in triplicate.

**FIGURE 1:**
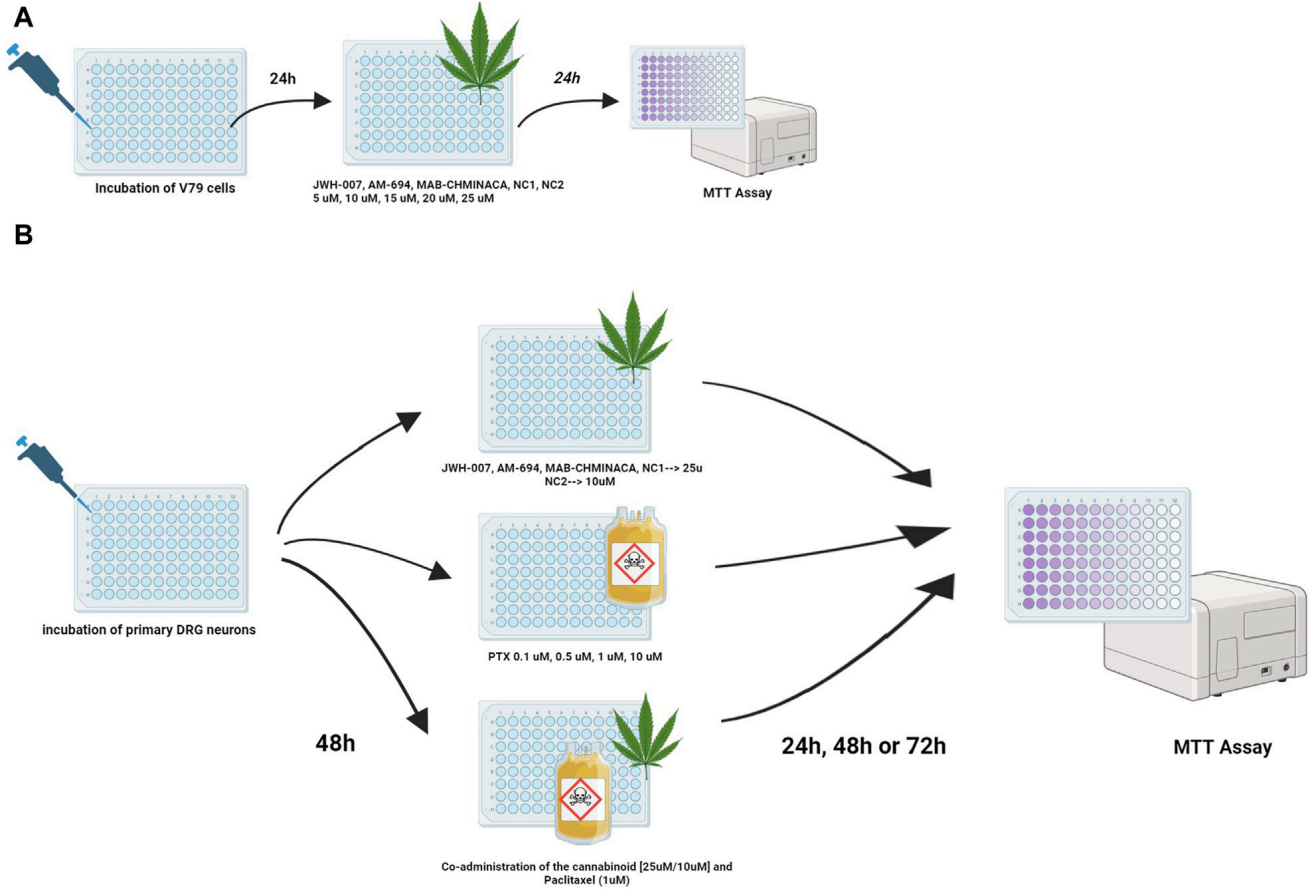
Treatment protocols for studying the viability of the cannabinoids and/or PTX on **(A)** fibroblasts or **(B)** DRG neurons were investigated using the MTT test. Viability was assessed at 24 h for fibroblasts and 24, 48, and 72 h post-treatment for neurons.

The viability of the cells was evaluated using a plate reader (EZ Read 400, Microplate Reader, Biochrom, UK), where optical densities were determined at 570 nm. The viability percentage was calculated as follows: cell viability percentage = (OD of treated cells/OD of untreated cells) × 100. Results are expressed as mean ± SD. The experimental design is presented in Figure 1.

#### 2.2.2 Neuronal toxicity and neuroprotection of the cannabinoids

##### 2.2.2.1 Primary neuronal culture establishing

DRG primary cultures are a suitable, simple, well-accepted method to assess the toxicity of pharmaceutical agents. Their ability to outgrow neurites *in vitro* and to react by decreasing the axonal length when in contact with neurotoxic agents makes DRG culture an excellent model for studying peripheral neuropathy induced by antineoplastic agents (Windebank et al., 1994). The presented method is adapted by Öztürk et al. (2013).

Swiss Albino female mice were deeply anaesthetised with isoflurane by inhalation followed by scarification. They were placed in a ventral position, and under aseptic conditions, the skin and soft tissues were removed along the vertebral column. Using a corneoscleral punch, the spinous apoptosis was crushed, and the spinal cord was exposed and scooped out. Parallel to the vertebral bodies, each costo-vertebral joint was cut using surgical scissors, clearing out the muscles, fat or other surrounding tissues. The dissection pieces were placed on ice, and using the stereomicroscope, DRGs were identified and collected from the intervertebral foramina on both sides. All collected DRGs were placed in a sterile dish containing the dissection media (RPMI-1640 with 1% Antibiotic/Antimycotic solution) until 15–20 were collected. After dissection, DRGs were moved to the culture media (Neurobasal A, 2% B27, 1% GlutaMax and 1% Antibiotic/Antimycotic solution), where Collagenase 1 mg/mL was added for the first enzymatic reaction (45 min at 37°C, 5% CO2).

At the end of the incubation, collagenase was removed by gently washing three times with 1 mL of HBSS. The second enzymatic reaction started with adding Trypsin EDTA at 0.25% for 15 min at 37°C, with 5% CO2. Soon after, 10 µL DNAse was added to the same tube for the third enzymatic reaction, and DRGs were gradually triturated for approximately 10 min with 1,000 µL pipette tip, 200 µL pipette tip and finally with insulin needle until the tissue was homogenised, followed by another incubation of 30 min. The enzyme activity was stopped by the action of the trypsin inhibitor solution and then removed by centrifugation at 180 g for 3 min. The DRG neurons were collected between the 35% and 10% layers of the Percoll gradient, washed with warm culture media and separated from debrides by centrifugation at 180 g for 20 min 24 h before harvesting, 12-well plates were pre-coated with 1 mg/mL Poly-D-lysine and 0.1 mg/mL laminin overnight at 4°C for the adhesion and neurite elongation of the cultured neurons. They were washed once with distilled H_2_O before seeding the cells in a culture medium. Separated neurons were seeded onto the centre of the coated coverslips for 2 h in an incubator with 37°C and 5% CO2, followed by adding the warm culture medium. The growth and morphology of neurons were monitored after 24 and 48 h.

##### 2.2.2.2 Neuronal toxicity

DRG neurons (9,000–10,000/well) were incubated for 48 h in 96-well plates and treated with different concentrations of Paclitaxel (0.1 µM, 0.5 µM, 1 μM, and 10 µM) and JWH-007 (25 µM), AM-694 (25 µM), MAB-CHMINACA (25 µM), NC1 (25 µM) and NC2 (10 µM) either individually or simultaneously combined to study the effects on cell viability. For the co-administration of the cannabinoids and PTX, the 1 µM dosage was chosen. Cell viability (%) was measured at 24 h, 48 h or 72 h using the abovementioned MTT assay. DRG neurons cultured in normal media free of PTX or/and the cannabinoids were used as control groups. Controls contained the similar highest concentration of DMSO (0.1%) to exclude any solvent effects on cell viability. All experiments were performed in duplicate. The experimental design is presented in Figure 1.

##### 2.2.2.3 Neurite preservation efficacy tests

**FIGURE 2:**
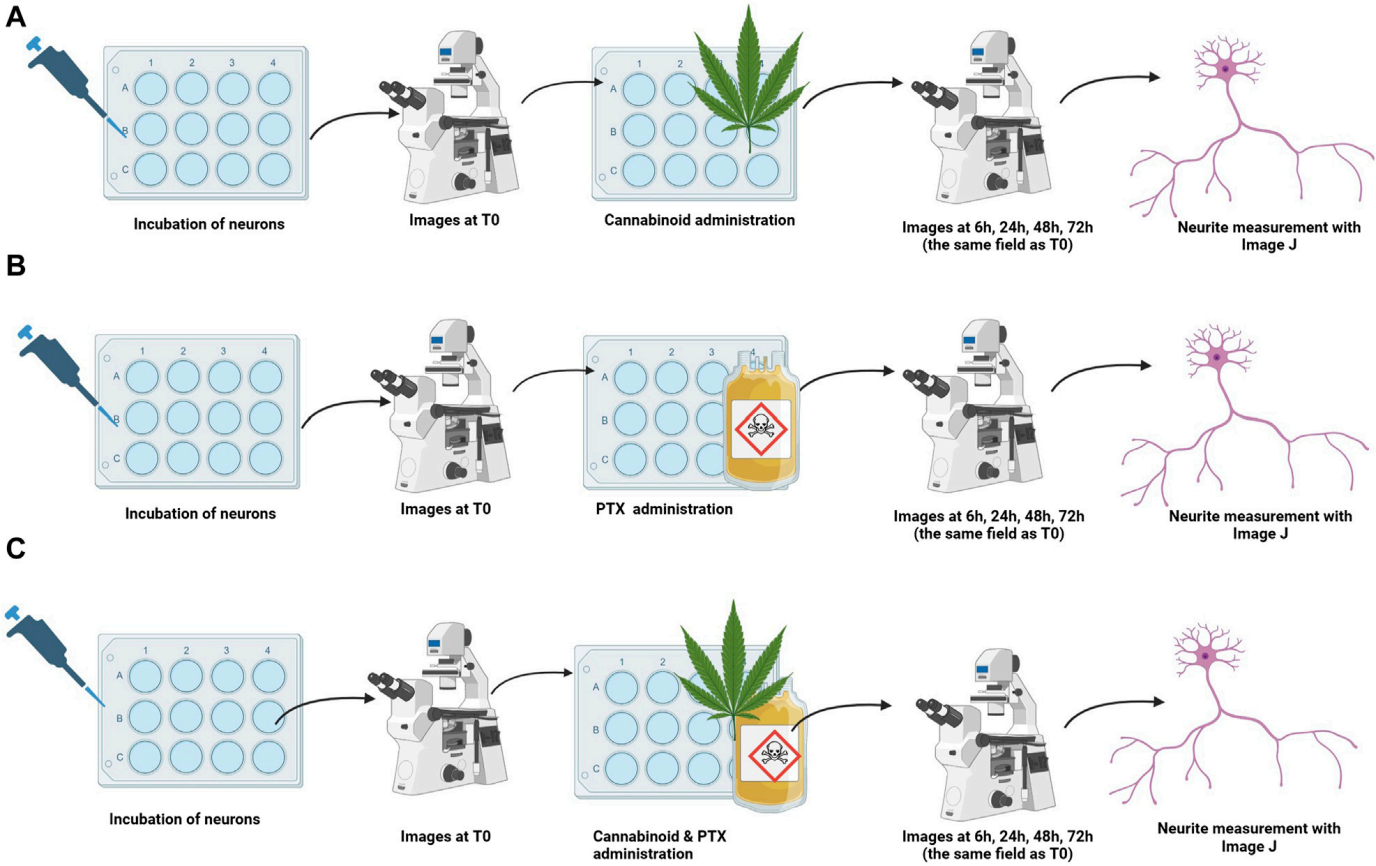
Treatment protocols for studying the efficacy of cannabinoids for neuroprotection. Neurons were incubated, and baseline images were taken. Treatments were administered **(A)** cannabinoid monotherapy, **(B)** PTX monotherapy, and **(C)** co-administration of the cannabinoids and PTX; neuron’s overall morphology and neurite length of the same microscopical field identified at T0 were assessed at 6 h, 24 h, 48 h, and 72 h after treatment administration.

The cannabinoid’s neuroprotective efficacy was assessed in three different settings (Figure 2): i) Assessment of the toxic/protective effects of each cannabinoid administered in monotherapy; ii) Determination of the toxic dose of PTX that induced shortening in the axonal length; iii) Assessment of the effects of the combination between each cannabinoid and PTX.

After complete adhesion, maturing and neurite elongation of the cultured neurons was observed, pictures of different microscope fields were taken using a microscope camera (Mshot, CMOS camera MD50-T). The corresponding place on the plate was marked using a fine-tip marker. Immediately after, treatments were administered. To identify the neurotoxic dose of the PTX, neurons were treated with different concentrations (0.1 µM, 0.5 µM, 1 µM, and 10 µM). These dosages were chosen based on previously reported studies (Guo et al., 2017; Akin et al., 2021; Park et al., 2021). The cannabinoid’s neuroprotective features when administered in monotherapy were tested by the addition of 25 µM of the SCs and NC1 or 10 µM of the NC2 on top of the cultured neurons and incubated at the same physiological conditions as mentioned before. We chose these dosages based on our preliminary findings regarding general fibroblast and neural toxicity. 6 h, 24 h, 48 h, and 72 h after the treatment administration, the same microscopic fields identified at T0 were monitored, and pictures were again taken.

For the co-administration of cannabinoids and PTX, fully matured neurons were simultaneously treated with either JWH-007 (25 µM), AM-694 (25 µM), MAB-CHMINACA (25 µM), NC1 (25 µM) or NC2 (10 µM) combined with1µM Paclitaxel and incubated at 37°C, 5% CO2. We chose this dosage of PTX, as we observed that it induced the highest neurite shortening while still keeping the targeted neurons viable at 48 h. As for the previous tests, images of the same neurites were obtained before any treatment and 6 h, 24 h, 48 h, 72 h after administration. For each set, neuronal death was qualitatively assessed and appreciated by the total disappearance of the neuron’s cellular body and neurites in the targeted microscopic field. The media of the controls contained either 1 µM of PTX or normal culture media with a similar concentration of DMSO (0.1%) used for treatment formulation to exclude any solvent effects on cell viability. Data comes from three independent experiments performed with three replicates, where five to eight microscopic regions were randomly recorded per plate, each field containing 1 to 5 measured neurons.

##### 2.2.2.4 Neurite tracing using ImageJ

The neurotoxicity of the treatments was assessed by measuring the neurite length of the targeted neurons. To calibrate distances, we followed the instructions provided by Pemberton et al., where an image with a known scale was used. The hemocytometer was chosen as a tool for calibration, as it has a known distance of 200 μm between two adjacent lines (Pemberton et al., 2018). Briefly, a sample image from treated DRG neurons was opened in ImageJ, (v1.46r (National Institutes of Health, Laboratory for Optical and Computational Instrumentation, University of Wisconsin, Madison, WI, United States) subtracted the background, converted to 8-bit grayscale and then individually opened in Neurite Tracer plugin. Each axon was semi-manually traced, and the lengths were automatically calculated. In addition, to ensure consistency and track temporal changes across the experimental timeline, measurements were taken at 6 h, 24 h, 48 h, and 72 h post-administration from the same fields and, consequently, the same neurons as those initially recorded.

##### 2.2.2.5 Statistical analysis

For the effects of the cannabinoids on the viability of the cells, results are expressed as %cell viability relative to controls (untreated cells) and represent the mean ±SD of two or three independent experiments. The difference from the controls was assessed using the one-way ANOVA test; a *p*-value<0.05 was considered significant.

The neuroprotective effects of the treatments were assessed relative to the baseline neurite length (T0). The analysis was focused on the axon shortening percentage for each measurement time (6 h, 24 h, 48 h, and 72 h), computed as the percentage change from the baseline for the neurite length of DRG neurons. Each neuron was compared to its baseline values. Data comes from two independent experiments performed with four replicates, where five to eight regions were recorded randomly per coverslip, each microscopic field containing 1 to 5 measured neurons. The normality of the data was evaluated using the Shapiro-Wilk Normality Test. Furthermore, Levene’s test for homogeneity of variances was conducted, and significant differences between groups were observed. A two-way ANOVA analysis was performed further to investigate the treatments’ significance on neurite outgrowth, followed by pairwise comparisons using T-tests. Bonferroni adjustment was applied to the *p*-values to address the issue of multiple comparisons. Additionally, pairwise comparisons were conducted assuming unequal variances, necessitating the Welch (or Satterthwaite) approximation to determine the degrees of freedom. *p*-value<0.05 was considered significant.

## 3 Results

### 3.1 Cannabinoid toxicity

**FIGURE 3:**
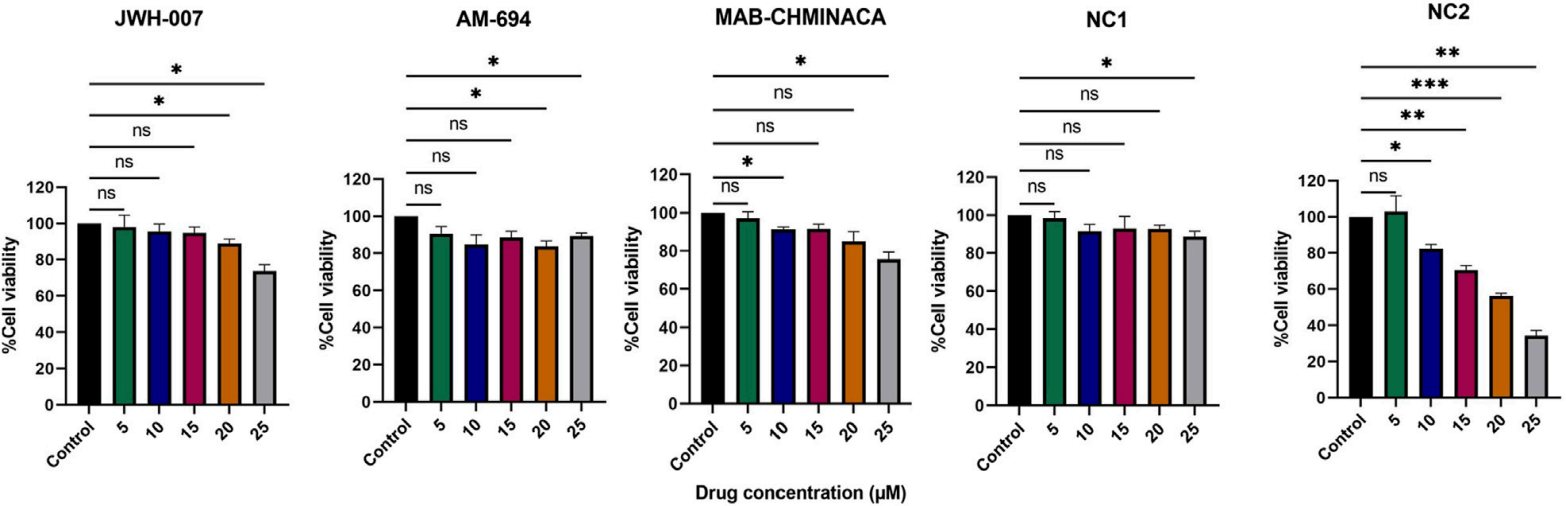
Effects of different concentrations of the cannabinoids (5, 10, 15, 20, and 25 µM) on viability (%) of the V79 fibroblast cell line, relative to controls (fibroblasts in normal culture media). Data is expressed as mean ± SD, performed in triplicate using the MTT assay (*p < 0.05, **p < 0.01, ***p < 0.001, ns = no statistical significance).

Fibroblast cells (V79 cell line) were treated for 24 h with different concentrations (5 µM, 10 μM, 15 μM, 20 μM, and 25 µM) of each of the cannabinoids. We found that the percentage of the viable cells varied with the dose and the type of cannabinoid administered (Figure 3). Each synthetic cannabinoid and NC1 had a good toxicity profile; even at the highest dosages, the viability of the cells remained higher than 70%, as seen in Figure 1. On the other hand, NC2 preserved the fibroblasts viability above 70% only for dosages lower than 10 µM. An additional 5 µM was conducted to a viability of 68%; in the case of the maximum tested dosage, 25 μM, the viability of the cells was 36%.

**FIGURE 4:**
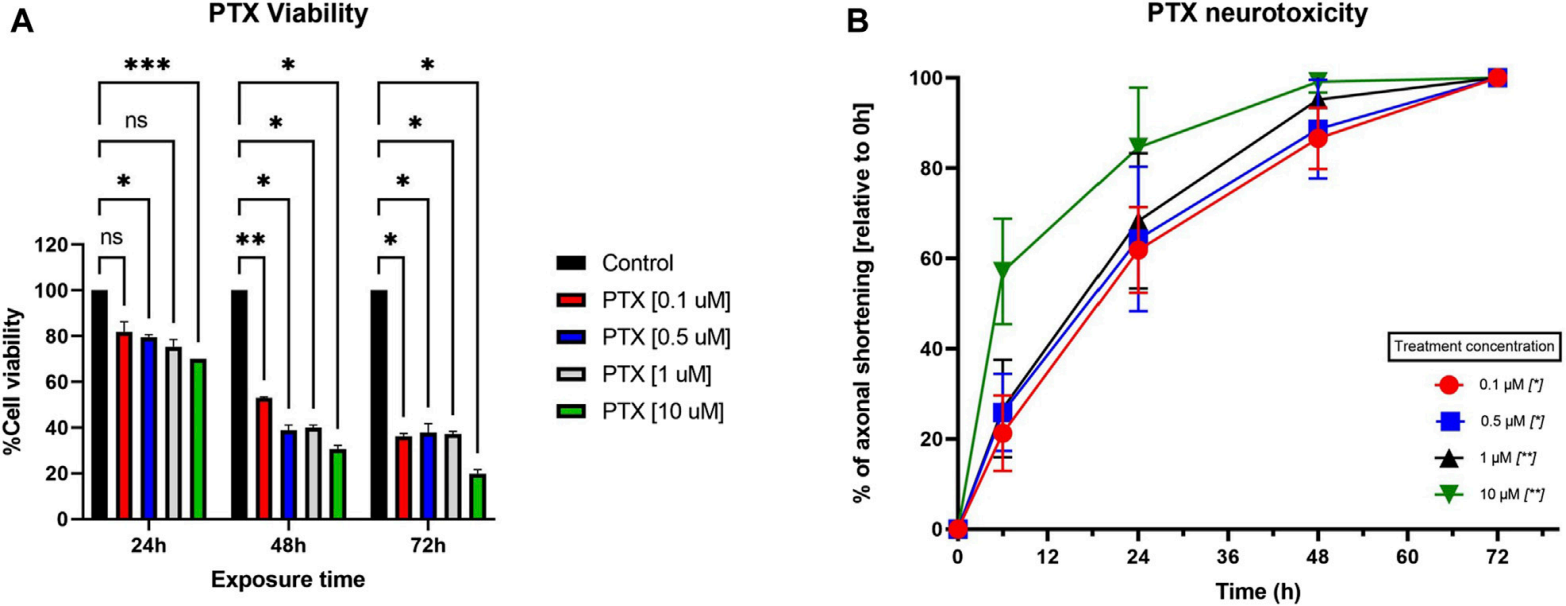
**(A)** Effects of different concentrations of PTX (0.1 µM, 0.5 µM, 1 μM, and 10 µM) on the viability of neurons, assessed with MTT. The results are expressed as % of viable cells and were measured at 3 different time points (24 h, 48 h, and 72 h post-administration). Data is expressed as mean ± SD; the results come from three different experiments performed in duplicate (*p < 0.05, **p < 0.01, ***p < 0.001, ns = no statistical significance). **(B)** Effects of different Paclitaxel concentrations (0.1 µM, 0.5 µM, 1 μM, and 10 µM) on neurite shortening of DRG neurons, measured at 6 h, 24 h, 48 h, and 72 h after treatment, compared to their initial lengths. Different concentrations of Paclitaxel had toxic effects leading to a reduction in neurite length, starting as soon as 6 h, compared to baseline values. Data is expressed as % of axonal shortening relative to each neuron’s baseline value and comes from three independent experiments performed with three replicates, where five to eight regions were recorded randomly per coverslip, each microscopic field containing 1–5 measured neurons (*p < 0.05, **p < 0.01, ns = no statistical significance).

**FIGURE 5:**
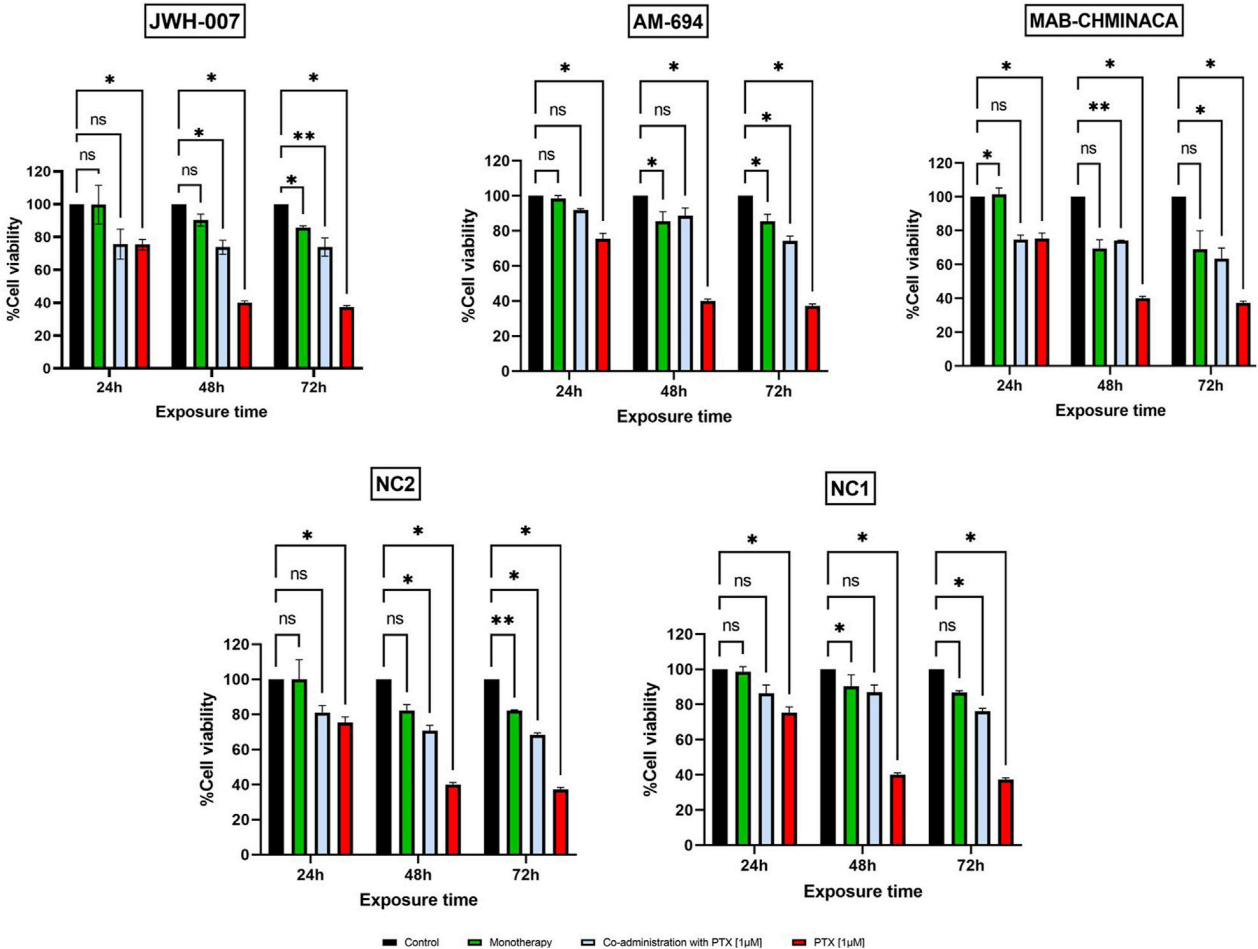
Effects of JWH-007 (25 µM), AM-694 (25 µM), MAB-CHMINACA (25 µM), NC1 (25 µM) and NC2 (10 µM) on neuron viability in monotherapy or co-administered with PTX (1 µM The results are expressed as % of viable cells and were measured at 3 different time points (24 h, 48 h, and 72 h post administration). Data is expressed as mean ± SD; the results come from three different experiments performed in duplicate (*p < 0.05, **p < 0.01, ns = no statistical significance).

When looking at the toxicity evaluated on primary neuron culture, 10 µM Paclitaxel had a clear cytotoxic effect on the cells at all tested time points, with viability dropping to less than 20% at 72 h post-administration, as seen in Figure 4A. On the other hand, the 0.1 µM, 0.5 µM, and 1 µM had cytotoxic features on neurons in a time-dependent, but not concentration-dependent manner, more robust starting with 48 h post-administration. The cannabinoids, as expected, showed no important effects on the viability of the cells when administered in monotherapy, except for MAB-CHMINACA, which conducted a cell viability of almost 70% 72 h post-administration. Combining the cannabinoids and PTX (1 µM) resulted in a clear difference in terms of % of viable cells, more importantly at 48 h and 72 h, where all tested cannabinoids preserved the survival of the neurons compared to chemotherapy alone (Figure 5).

### 3.2 Cannabinoid efficacy

#### 3.2.1 Establishing of the primary DRG neuronal cells

**FIGURE 6:**
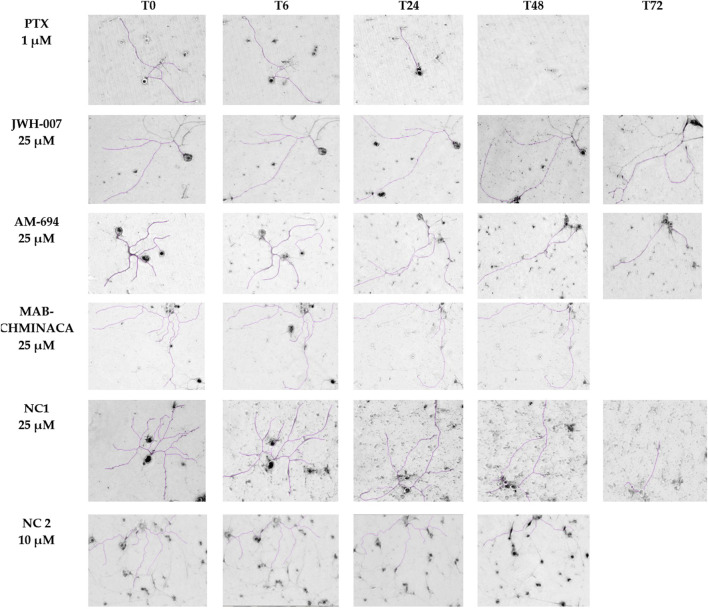
Neurons’ morphological features and neurite outgrowth measurements with ImageJ. On each row, the time-dependent effects of each compound administered in monotherapy are presented, scale bars 200 µm.

Established primary DRG neuron cultures were examined under a phase-contrast microscope at various time points (before treatment (T0) and at 6, 24, 48, and 72 h after treatment) to analyse the overall cellular morphology and track the axonal length. At each time point, the same microscope field was analysed. At T0, all neuron somas appeared round, bright, and refractile, with a large nucleus. The neurons had long, extended, thin neurites that connected and formed networks. Afterwards, due to the action of the treatments, the neurites’ lengths began to modify in a time-dependent manner, as seen in Figure 6.

#### 3.2.2 Paclitaxel-induced neurotoxicity

PTX’s toxic effects on the neurites were visible starting from the first assessment (6 h), with total neuronal death at 72 h post-administration, as observed in the targeted microscopic fields, regardless of the dose (Figure 4B). In the case of 0.1 µM and 0.5 µM PTX, 24 h after the treatment administration, more than half of the initial axonal length was lost, as seen in Figure 3. 48 h after treatment initiation, cellular death appeared for 13% and respectively 25% of the targeted neurons. The 10 µM concentration had the most toxic effect on the cells; at 24 h, only 63% of the neurons were still viable, and the remaining ones had a preserved neuronal length of almost 20% of their initial values. In the case of the neurons treated with 1 μM, we saw a time-dependent axonal shortening, with the most balanced proportion between maximal axonal shortening and cellular death. Compared to the initial values, the percentages of the remaining axons varied between the ones of the 0.5 µM and 10 µM treated groups at each time point tested. Following the same trend as the rest of the treated groups regarding neuronal death, only 25% of the cells were still identifiable on the targeted microscopic fields 48 h after treatment administration.

#### 3.2.3 JWH-007

##### 3.2.3.1 Monotherapy

**FIGURE 7:**
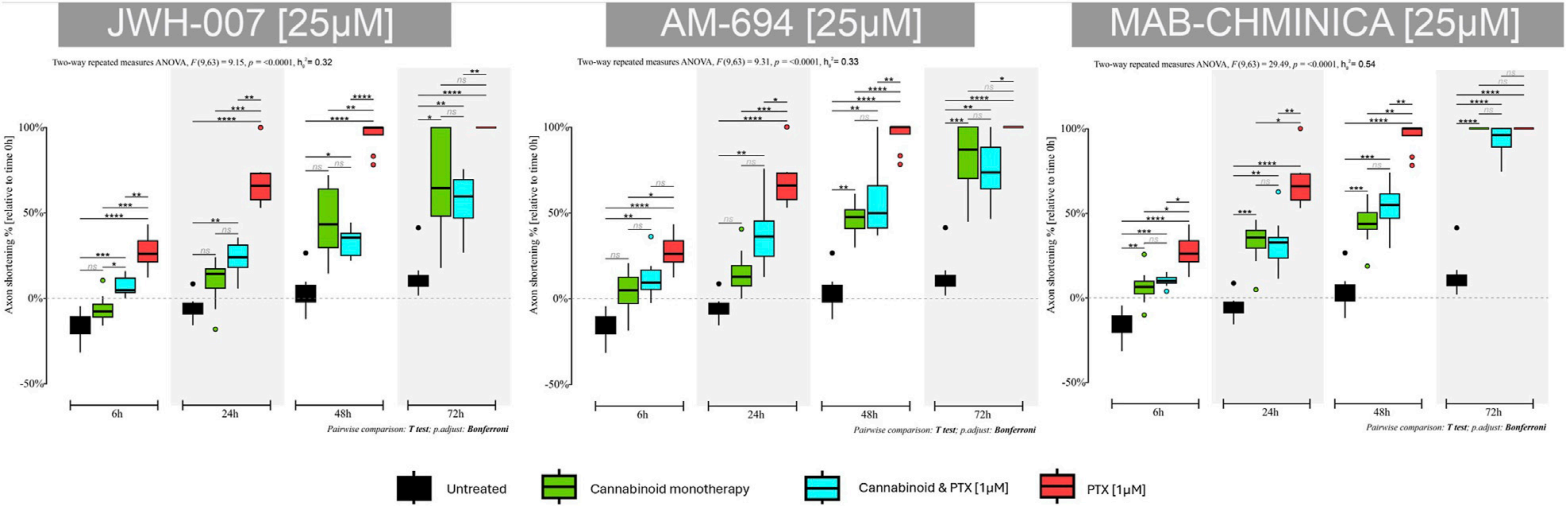
Effects of either 25 µM JWH-007, 25 µM AM-694, 25 µM MAB-CHMINACA on axonal lengths of DRG neurons, measured at 6 h, 24 h, 48 h, and 72 h after treatment, either alone or combined with 1 µM PTX. Data is expressed as % of axonal shortening relative to each neuron’s baseline value. Controls are neurons in normal media and neurons treated with 1 µM PTX. Data comes from two independent experiments performed with four replicates, where five to eight regions were recorded randomly per coverslip, each microscopic field containing 1 to 5 measured neurons. (*p < 0.05, **p < 0.01, ***p < 0.001, ****p < 0.0001, ns = no statistical significance).

JWH-007 had a neuroprotective effect as soon as 6 h after administration, with an almost 6% increase in neurite length (Figure 7). However, this neurotrophic effect was soon over, at the 24 h timepoint the neurites began to shorten compared to untreated neurons that continued to elongate their neurites with an additional 10% from the initial values. In contrast to the known neurotoxic agent PTX, this shortening effect was significantly lower (*p* < 0.0001) at 24 h, with neurites preserving 91% of their initial length, compared to the PTX group, which preserved 32%. This effect (*p* < 0.0001) was preserved for the T48 time-point. Interestingly, 72 h after the treatment initiation, neurons were still viable, and the axons were visible and measurable.

##### 3.2.3.2 Combination therapy

Adding PTX to the JWH-007 solution was associated with a more pronounced shortening of the axons than monotherapy but still significantly better than PTX alone (*p* < 0.001), as seen in the 24-h timepoint. Paradoxically, 48 h and 72 h into the treatment, the combination led to better neurite preservation than cannabinoid monotherapy (65% vs. 55% at 48 h); however, it was not statistically significant.

#### 3.2.4 AM-694

##### 3.2.4.1 Monotherapy

AM-694 induced neurotrophic activity 6 h after the treatment administration, with a downward trajectory soon after (*p* < 0.01) (Figure 7). As with the JWH-007, the % of axonal shortening was significantly lower compared to the PTX-only group (*p* < 0.001 at 24 h, *p* < 0.0001 at 48 h). At 72 h, neurons remained viable and had a preserved neurite length of 18% from the initial value. However, the untreated neurons kept 87% of their initial length.

##### 3.2.4.2 Combination therapy

The addition of PTX to AM-694 accelerated the neurite shortening, preserving the axonal length of 63% and 43% for the combination group compared to the 84% and 53% of the monotherapy at 24 h and 48 h. Still, the combination was much better than PTX in terms of axonal length preservation (*p* < 0.01 at 24 h, *p* < 0.001 at 48 h and *p* < 0.01 at 72 h). The same paradoxical effect of better combination effects compared to monotherapy was seen in this group of AM-694 treated neurons at 72 h post-therapy initiation.

#### 3.2.5 MAB-CHMINACA

##### 3.2.5.1 Monotherapy

MAB-CHMINACA induced axonal shortening at all tested time points; however, at 24 h, it reduced the lengths by 22% compared to the PTX group and their 68% shortening (*p* < 0.01) (Figure 7). The same effect was seen at 48 h (*p* < 0.01), in contrast to the untreated neurons that preserved 95% of their initial values—also, the cannabinoid-induced cellular death at 72 h post-treatment initiation, similar to the PTX.

##### 3.2.5.2 Combination therapy

The coadministration of MAB-CHMINACA and PTX was similar in axonal shortening compared to the monotherapy; however, it was better than PTX alone. At 24 h, 67% of the initial neurite’s length was preserved, compared to 78% in the monotherapy and 32% in the PTX group (*p* < 0.01). Similarly, at 48 h, more of the neurite length was preserved compared to the PTX group (*p* < 0.01).

#### 3.2.6 NC1

##### 3.2.6.1 Monotherapy

**FIGURE 8:**
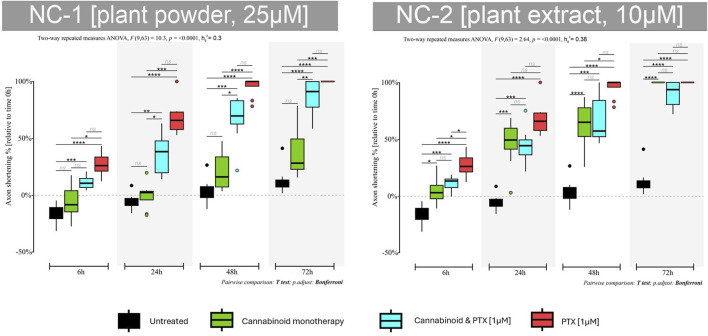
Effects of either 25 µM NC1 or 10 µM NC2 on axonal lengths of DRG neurons, measured at 6 h, 24 h, 48 h, and 72 h after treatment, either alone or combined with 1 µM PTX. Data is expressed as % of axonal shortening relative to each neuron’s baseline value. Controls are neurons in normal media and neurons treated with 1 µM PTX. Data comes from two independent experiments performed with four replicates, where five to eight regions were recorded randomly per coverslip, each microscopic field containing 1 to 5 measured neurons (*p < 0.05,**p < 0.01, ***p < 0.001, ****p < 0.0001, ns = no statistical significance).

When administered alone, the NC1 phytocannabinoid had the highest neuroprotective effect at 6 h and 24 h (Figure 8). The neurite’s length increased in total size by 7% compared to their initial values (*p* < 0.01). More prolonged exposure led to axonal shortening, but the lengths were significantly higher than in the case of PTX. 79% of the axonal length was preserved at 48 h compared to 95% in the untreated group and 3% in the PTX.

##### 3.2.6.2 Combination therapy

When adding the neurotoxic agent, some of the neuroprotective features of the NC1 were obviously lost. However, the cannabinoid protected the neurites from accelerated shortening, and the axons preserved 63% of their initial lengths at 24 h, compared to 32% in the PTX-treated group (*p* < 0.001) and 33% at 48 h vs. 3% (*p* < 0.001). Moreover, neurons were still viable at 72 h and also preserved 14% of their initial axonal length.

#### 3.2.7 NC2

##### 3.2.7.1 Monotherapy

NC2 induced axonal shortening at all tested time points, but the results were much better than those of the PTX group (Figure 8). The axons shortened by 46% at 24 h compared to the PTX group that lost 32% of their length (*p* < 0.01). The same trend was seen at 48 h (*p* < 0.01)—moreover, the phytocannabinoid induced cellular death at 72 h after treatment initiation, similar to PTX.

##### 3.2.7.2 Combination therapy

NC2 protected the neurons from the toxic effects of the PTX. Combining the two agents caused neurites to shorten less than when using PTX only. This group had a 55% and 32% preserved axonal length at 24 h and 48 h compared to 32% and 3%, respectively.

## 4 Discussion

Phytocannabinoids include more than 110 natural compounds, with more than 100 lipophilic molecules identified in their structure, Δ9-THC and CBD being the best-known representatives. Historically, synthetic cannabinoids have been synthesised to localise CBRs, being 2-100 times more potent than Δ9-THC, even though they are similar in chemical structure. Because of the increased risk of abuse and serious side effects, SCs became overlooked for their medicinal benefits (Tamba et al., 2020).

Given the immense variability in composition, thorough safety and efficacy tests must be conducted if translation of the cannabinoids in the clinic is to be achieved. In our study, the tested cannabinoids had an acceptable toxicity profile on fibroblasts and neurons. Even at the highest tested dosages, the SCs and NC1 showed no important cytotoxicity in fibroblast toxicity. NC2 had a good toxicity profile up to the 10 µM dose, and then the viability of the fibroblasts dropped below the 70% threshold. When measuring the cytotoxicity of the compounds on neurons, our data showed that PTX had a toxic effect on the viability of the cells in a time-dependent manner but concentration-dependent for only the 10 µM dosage, in line with similar studies (Guo et al., 2017; Elfarnawany and Dehghani, 2022; Elfarnawany and Dehghani, 2023). However, when PTX was combined with the cannabinoids, the percentage of cytotoxicity decreased, suggesting a protective role that cannabinoids have for preserving neurons. In addition, the viability of the neurons was qualitatively assessed, along with the neurite outgrowth measurements. We could observe that the intense toxicity of PTX was conducted not only to axonal shortening but also to complete neuronal death (the disappearance of the targeted cell), as opposed to the cannabinoids. We could see the same effect as observed with the MTT assay; the addition of cannabinoids on the PTX-treated neurons was conducted to prolong the survival of the cells. Notably, the best results were observed with JWH-007, AM-694 and NC1, with the lowest neuron viability being more than 65% at 72 h after the treatment.

Advances in neuropathic pain therapies are challenging due to the ethical and practical difficulties of pain studies in the human population. Thus, preclinical models are commonly used to mimic similar pathological modifications encountered in neuropathic pain. Moreover, establishing relevant preclinical neuropathic pain models allows the development of clinically efficient pain prophylactic drugs (Sousa et al., 2016). High concentrations of PTX were found to accumulate in the dorsal root ganglia (DRG) and not in the peripheral nerves, suggesting that changes in the peripheral nerve fibres may be secondary to DRG neuron involvement (Cavaletti et al., 2000). Animal studies are often used to investigate the systemic modifications produced by neuropathic pain and the effects of different therapies on peripheral nerves. Still, they are only sometimes suitable to study local interactions. Therefore, various *in vitro* culture systems have been developed to understand the mechanisms of neurotoxicity/neuroprotection. Primary neuronal cell culture is commonly employed in research to retain the cell’s original structure and physiology (Amini and White, 2013).

Up to 68.1% of cancer patients treated with Paclitaxel develop peripheral neuropathy (Klein and Lehmann, 2021). Although neurons are not dividing cells, they are also a target of PTX’s toxicity, with DRG neurons highly susceptible to chemotherapeutic drug accumulation (Cavaletti et al., 2000). In the current study, PTX caused axon shortening, thus neurotoxicity of the cultured neurons in a dose- and exposure-time-dependent manner, which aligns with similar studies (Carlson and Ocean, 2011; Jaggi and Singh, 2012). The toxic effects of PTX were rapid, with a significant reduction in neurite length of neurons as early as 6 h post-treatment administration. The toxicity is caused by the accumulation of the drug at the neuronal level, which leads to higher susceptibility or vulnerability of neurites to toxins than neuronal somata (Scuteri et al., 2006; Guo et al., 2017).

Reports of the neuroprotective activity of cannabinoids were published decades ago when various cannabinoids were shown to exert neuroprotection upon several disease models such as Parkinson’s Disease, Alzheimer’s Disease, Multiple Sclerosis, and, nevertheless, CIPN (Sarne and Mechoulam, 2005; Galve-Roperh et al., 2008). Using cannabinoids as drugs that target neuroprotection makes them potential candidates to reverse or prevent the neurodegenerative processes that underlie neuropathic pain. To mention a few examples exploring this indication, synthetic cannabinoid WIN-55,212-2, a mixed CB1R/CB2R-receptor agonist, was tested on DRG neurons, and authors found significant results for neuropathic pain modulation through the inhibition of the TNF-α-induced expression/activity of NOS in the treated neurons (Tan and Cao, 2018) The same WIN-55,212-2 confers neuroprotection in a model of neonatal hypoxic-ischemic encephalopathy (Fernández-López et al., 2006) and protects the rat hippocampal neurons from excitotoxicity (Blanton et al., 2019). Incubation of DRG neurons with cisplatin URB597 and JZL184, inhibitors of the endocannabinoid 2-arachidonoylglycerol (2-AG) hydrolysis, attenuated the neurite shortening changes through activation of CB1R (Khasabova et al., 2011; Khasabova et al., 2012). Also, THC administration reduces neuronal loss and brain damage in excitotoxicity and ischemia models (van der Stelt et al., 2001), while 2-arachidonoylglycerol (2AG) protects neurons in traumatic brain injury (Panikashvili et al., 2001).

DRG neurons have diversified morphology and functions responsible for neuropathic pain signal transduction and modulation by the expression of various ion channels and receptors (Berta et al., 2017). The alterations caused by PTX were commonly observed in the transient receptor potential channels (TRP), voltage-gated ion channels, glutamate, and ATP-sensitive receptors in the DRG neurons (Krames, 2014). The molecular mechanisms underlying the neuroprotective action of cannabinoids are related to the direct action of ligands on the endocannabinoid system by activating CB1R and CB2R (Ramírez et al., 2005). Other structures involved are the nuclear receptors of the PPAR family, transcription factors, serotonin 1A receptors and the adenosine signalling pathway (Antonazzo et al., 2019).

Agonists of the CB1R can provide neuroprotective effects by normalising glutamate homeostasis, as the receptors are located at neuronal glutamatergic terminals, and their activation reduces glutamate release (Mechoulam et al., 2002). Moreover, cannabinoids can modulate the activity of voltage-sensitive calcium channels, which consequently could diminish the calcium influx (Demuth and Molleman, 2006). Another neuroprotection mechanism is restoring the equilibrium between oxidative and antioxidant mechanisms within the neurons. It is related to the cannabinoid’s ability to reduce excessive production of the reactive oxygen species by acting as scavengers and antagonising lipid peroxidation (Chen et al., 2000).

Our results clearly show that cannabinoids offer neuroprotection in delaying or preventing neurite shortening, the marker for neuronal suffering. This effect is shown in all the tested cannabinoids when administered together with the PTX. The cannabinoids seemed to be reversing part of the damage that PTX does, with the neurite lengths being significantly longer when a combination of the drugs was administered. When assessing the neurites length of the cannabinoids administered in monotherapy, compared to the untreated controls, we could, in fact, see that they have a toxic impact on their own when administered for more than 24 h. Only two of our tested cannabinoids, JWH-007 and the phytocannabinoid NC1, had a neurotrophic effect in terms of stimulating the axonal elongation, similar to the untreated neurons, but this effect was emphasised only briefly. However, the toxicity of the cannabinoids administered in monotherapy is incomparable with the damage that PTX does.

This “yin-yang” feature of neuroprotection/neurotoxicity of cannabinoids present in our study has also been identified by other research groups. It has been shown to be highly dependent on experimental factors. The dual (neurostimulator and neuroinhibitory) effects of cannabinoids depend, in most cases, on the concentration of the drug tested. Regular (high) concentrations induced the conventional inhibitory (neuroprotective) effects, and low concentrations of cannabinoids induced stimulatory (neurotoxic) effects (Sarne and Keren, 2004). Moreover, short-time exposure to the cannabinoids results in a high concentration of the drug on neurons and, therefore, will protect the cells from damage, while longer exposure times promote nerve damage (Lawston et al., 2000; Panikashvili et al., 2001; van der Stelt et al., 2001). Although *in vitro* studies are suitable for assessing the local effects of cannabinoids, neuroprotection does not rely solely on neurons but also on glial cells or vascular endothelium, which is why cannabinoid’ neuroprotection is sometimes more evident *in vivo* studies (Guzman, 2003). Some results worth mentioning are WIN 55,212-2, which has been found to reduce cold allodynia and thermal hyperalgesia symptoms with only one administration on a nerve ligation neuropathy murine model (Rahn et al., 2007). CBD has also demonstrated an antinociceptive effect in mechanical and cold allodynia induced by CIPN using paclitaxel (Ward et al., 2011; Ward et al., 2014). Mixed agonists (∆9 -THC, CP55,940, and WIN55,212-2) reversed mechanical and cold allodynia and plantar heat hyperalgesia in CIPN models (Rahn et al., 2014; Deng et al., 2015a; Deng et al., 2015b).

One interesting finding in our study was that after 48 h of treatment, monotherapy with the SCs caused a higher shortening of the axons compared to the combination of the cannabinoids and PTX. One explanation could be found in the mechanisms mentioned earlier; however, another possibility could be seen when looking at the similarities between the cannabinoids and PTX mechanisms of action. In the case of PTX, besides the activity on the microtubules, the mitochondrion has been put forth as a potential mediator of toxicity due to its ability to alter its structure and function. Studies have shown that PTX evokes immediate mitochondrial depolarisation, and due to the opening of the mitochondrial permeability transition pore, it induces dysregulation of the axonal transport through the voltage-gated calcium channels (Gornstein and Schwarz, 2014). Also, mitochondrion is important in regulating intracellular calcium homeostasis, where increased calcium oscillations are observed after PTX treatment (Kidd et al., 2002). Consequently, the changes that PTX induces in the mitochondrial structure are related to increased calcium-mediated neuronal excitability (Jaggi and Singh, 2012). On the other hand, the interaction of the cannabinoids with G-coupled receptors on the neuronal surface attenuates the cAMP production, which causes a reduction in neuronal activity by modulating the potassium channels and inhibiting voltage-gated calcium channels (Howlett et al., 2010). The modulation of the voltage-dependent calcium channels reduces the elevation of intracellular calcium and, consequently, the release of glutamate, as described in previous paragraphs (Mechoulam et al., 2002; Demuth and Molleman, 2006). On the contrary, some cannabinoids potentiate the internalisation of calcium; thus, high levels of intracellular calcium initiate a complex cascade of intracellular events, such as the stimulation of various proapoptotic enzymes, which affect cell homeostasis and lead to paradoxically, neuronal suffering (Sarne and Mechoulam, 2005). These common mechanisms of action between cannabinoids and PTX could raise the hypothesis of whether there is a non-competitive/competitive antagonistic relationship between the drugs, causing the cannabinoids to temperate part of the harmful action of the chemotherapeutic drug. However, this hypothesis needs further exploration.

When comparing the effects of the best candidate from the SCs, JWH-007 and the best phytocannabinoid, NC1, we observed better effects for preserving the neurite length of the natural cannabinoid when administered in monotherapy. Each strain of cannabis plant can have variations in the concentration of the substances within. It usually contains over 500 other chemical compounds such as cannabinoid phenols, non-cannabinoid phenols, alcohols, aldehydes, n-alkanes, alkaloids, flavonoids, terpenoids, wax esters and steroids, which in turn may modulate the effect of cannabinoids, the so-called “entourage effect” (Ben-Shabat et al., 1998). All these molecules are believed to have synergistic interactions and not only enhance the cannabinoid’s activity but also independently modulate some beneficial effects. Even though SCs are more potent, they do not benefit from the entourage effect, as the accompanying molecules are not incorporated during manufacturing. In contrast, this “simple” structure of JWH-007 was the key to better neuroprotective features when co-administered with PTX, as opposed to NC1, which showed more modest results, probably due to the unknown interactions of some of the plant’s components with the chemotherapeutic drug.

Based on our knowledge and literature search, these cannabinoids were never tested for their potential neuroprotective features and, consequently, analgesic effects for *in vitro* CIPN models. JWH-007 is a chemical from the naphthoylindole family, which acts as an agonist at both the CB1R, with a binding affinity of 9.5 nM and CB2R, with a binding affinity of 2.94 nM*. AM-694 and MAB-CHMINACA are CB1R agonists, but their binding affinity differs. AM-694* has a K_i_ of 0.08 nM at CB1R and 18 times selectivity over CB2R with a K_i_ of 1.44 Nm. *MA*B-CHMINACA is a potent agonist of the CB1R with a binding affinity of *K*_i_ = 0.289 nM and was initially developed by Pfizer^®^ in 2009 as an analgesic medication. However, no results regarding its efficacy have been published. The class of synthetic substances known as JWH contains over 100 compounds, all having a much higher affinity for the CBR than natural cannabinoids. Some of the representatives, JWH133 and JWH015, have been found to decrease mechanical allodynia in a nerve ligation neuropathic murine model (Romero-Sandoval et al., 2017). JWH-007s and AM-694s increased affinity for the CB1R could explain the better results obtained in our study regarding neuroprotection, in contrast to the others with lower affinity for the receptor. As for the phytocannabinoids, Cannabixir^®^ Medium dried flowers (NC1) are in the form of a dried inflorescence of Cannabis sativa L. and have a 15.6% THC and <1% CBD content. Cannabixir^®^ THC full extract (NC2), a full THCA-enriched extract of female Cannabis buds, contains ∼20% THC. Nevertheless, Δ9-THC is considered a mixed CB1R/CB2R agonist and has also been shown to alleviate pain, as observed in our study (Rahn et al., 2014; Deng et al., 2015a).

Our results contribute to the knowledge of the benefits that cannabinoids can have for oncological patients, with a particular focus on neuropathic pain. Using cannabinoids as agents that prevent or delay the installation of CIPN is a major contributor to the analgesia and, consequently, the quality of life of cancer patients. Moreover, preventing CIPN allows clinicians to use more aggressive therapeutic schemes, which would give better tumour control while minimizing the side effects, with significantly high input on patient survival. Altogether, neuroprotection of our tested cannabinoids is not a feature desirable only for neuropathic pain and analgesia of cancer patients. These neuroprotective effects could be translated to other models where damage to the peripheral neurons is implied, primarily neurological disorders. Our results are promising for a larger cohort of pathologies and could be further explored using various disease models.

Even though the presented results are promising, we acknowledge that our study isn’t without limitations. The neuroprotection of candidate drugs can be assessed in multiple ways, neurite length dynamics being the most used; however, it is not the only one available (Lehmann et al., 2020). More studies need to be conducted to evaluate the effects of cannabinoids on glial cells, vascular endothelium, and other structures involved in pathogenesis. Additionally, further studies need to investigate the mechanism of action of cannabinoids for neuroprotection. As neurotoxicity could be considered a syndrome due to the multiple mechanisms, cell types, receptors, and neurotransmitters involved, the *in vitro* studies must be followed by investigations of the effects in living organisms, contributing to the overall knowledge on the topic. Our study’s purpose is exploratory, as it is the first study to screen the chosen cannabinoids in the setting of paclitaxel-induced toxicity; of course, all experimental studies raise questions and hypotheses and must be followed by other mechanism-oriented studies.

## 5 Conclusion

Our study paves the way for the benefits of either synthetic cannabinoids or phytocannabinoids for the palliation of chemotherapy-induced peripheral neuropathy. We found that three synthetic cannabinoids (JWH-007, AM-694 and MAB-CHMINACA) and two phytocannabinoids (Cannabixir^®^ Medium dried flowers (NC1) and Cannabixir^®^ THC full extract (NC2)) had an acceptable toxicity profile on fibroblasts and primary neuronal culture and can be an effective option for paclitaxel-induced peripheral neuropathy. The synthetic cannabinoid JWH-007 and the phytocannabinoid Cannabixir^®^ Medium dried flowers (NC1) had the best results in their class. They presented good neurotrophic and neuroprotective activity on the primary dorsal root ganglion culture model. The present findings must be followed by additional tests to understand the exact mechanism of action better and further investigate these results in the *in vivo* setting. Cannabinoids are at a critical tipping point in science; however, there is a need for more high-quality basic science, which would ensure the successful translation of the cannabinoids into clinical trials.

## Data Availability

The raw data supporting the conclusion of this article will be made available by the authors, without undue reservation.

## Abbreviations

CIPN, chemotherapy-induced peripheral neuropathy; PC, phytocannabinoid; SC, synthetic cannabinoid; CBD, cannabidiol; THC, tetrahydrocannabinol; Δ9 -THC, delta-9-tetrahydrocannabinol; 2AG, 2-arachidonoylglycerol; CB, cannabinoid receptor; CB1R, cannabinoid receptor 1; CB2R, cannabinoid receptor 2; TRPV1, transient receptor potential vanilloid 1; PPARs, peroxisome proliferator-activated receptors; NC1, cannabixir^®^ medium dried flowers; NC2, cannabixir^®^ THC full extract; PTX, paclitaxel; DMSO, dimethylsulfoxide; DRG, dorsal root ganglion; GABA, γ-aminobutyric acid; TNF-α, tumor necrosis factor-α; NOS, nitric oxide synthase; cAMP, cyclic adenosine monophosphate; ESMO, European society of medical oncology; ASCO, American society of clinical oncology.
